# A comparison of physical activity and nutrition in young women with and without primary dysmenorrhea

**DOI:** 10.12688/f1000research.12462.1

**Published:** 2018-01-16

**Authors:** Dina Abadi Bavil, Mahrokh Dolatian, Zohreh Mahmoodi, Alireza Akbarzadeh Baghban

**Affiliations:** 1 Department of Midwifery and Reproductive Health, School of Nursing and Midwifery, Shahid Beheshti University of Medical Sciences, Tehran, Iran; 2 Non-Communicable Diseases Research Center, Alborz University of Medical Sciences, Karaj, Iran; 3 Proteomics Research Center, Department of Basic Sciences, School of Rehabilitation Sciences, Shahid Beheshti University of Medical Sciences, Tehran, Iran

**Keywords:** Nutrition, physical activity, dysmenorrhea, young women

## Abstract

**Background:** Dysmenorrhea is the most common gynecological disorder in young women and is seen in almost 50% of women. The present study was conducted to investigate the relationship between physical activity and nutrition with primary dysmenorrhea in students at Mazandaran University of Medical Sciences (Sari, Iran) in 2015.

**Methods:** This comparative descriptive study was conducted on 250 students with and without primary dysmenorrhea. Data were collected using personal-demographic, nutrition and physical activity questionnaires. The output was then analyzed in SPSS-18 using independent t-test, Chi-square test and logistic regression analysis.

**Results:** The results showed significant differences between the two groups in terms of nutrition and physical activity, as the mean score of nutrition was 57.91 in the group with dysmenorrhea and 61.68 in the group without, while the mean intensity of physical activity was 5518.75 metric in the group with dysmenorrhea and 4666.42 metric in the group without. Physical activity was calculated by MET scale (minutes/week). This index measured the amount of consumed energy at the time of activity relative to that consumed at resting time.

**Conclusions: **A healthier and more favorable nutrition style and more regular physical activity reduces the severity of dysmenorrhea in girls. Therefore, educational measures are required to raise awareness among young women about the effects of proper nutrition and physical activity on the prevention and reduction of dysmenorrhea complications.

## Introduction

Primary dysmenorrhea is one of the most common gynecological disorders that refer to cramping pain in the lower abdomen during menstruation without pelvic pathology. This complication often occurs in the first and second years after the onset of menstruation during ovulation ^1^. The overall prevalence of primary dysmenorrhea is 60% to 90% in adolescent girls but decreases with age ^2^. Increased concentrations of prostaglandins and vasopressin, increased levels of leukotrienes and psychological factors are reported to be involved in the development of primary dysmenorrhea ^3^. Prostaglandins cause pain by increasing uterine tone and contractions ^1^. There are several medicinal and non-medicinal methods for improving or eliminating this complication. A non-medicinal treatment for primary dysmenorrhea is changing nutrition; for instance, reducing the intake of salt and animal fats, increasing the consumption of complex carbohydrates and dietary fibers and increasing physical activity ^4^.

Although various treatment methods have been proposed for this complication, there has been limited success. Some studies have proposed factors such as dietary habits ^5^ nutrition ^6^ and aerobic exercise ^7^ as effective in the treatment of dysmenorrhea, but one study found no relationship between exercise and dysmenorrhea ^8^. Since medicinal therapies can have side-effects, and as some people prefer to not be medicated, researchers and young women are both seeking alternative therapies for this condition ^9^. The disparity of findings on this disorder led to the present study about nutrition and physical activity and their relationship to primary dysmenorrhea in university students, so as to facilitate interventions targeting nutrition and physical activity in young women.

## Methods

### Participants

The present comparative descriptive study was conducted on 250 female students at Mazandaran University of Medical Sciences (Sari, Iran). Students were recruited during lectures at the university. Students with menarche who had menstrual pain and without pelvic pathological disorders and this pain limited to menstrual periods were classified as primary dysmenorrhea, which was self-reported.

 Sampling lasted from late August to late November 2015. A total of 125 students belonged to the case group with primary dysmenorrhea and 125 students to the control group without this condition were case-matched to the study group through convenience sampling. The inclusion criteria for the cases consisted of being single, age 18 to 26, having moderate or severe (scores 4 to 10) and painless (scores 0 to 3) primary dysmenorrhea based on the McGill Pain Index, having no known chronic diseases, such as diabetes, hypertension, underlying cardiac diseases, infectious diseases, etc., having no self-reported symptoms such as burning, itching and abnormal vaginal discharge, and having no history of gynecological surgeries.

Sample size was calculated using the formula:

n=[z1−α22π¯(1−π¯)+z1–βπ1(1−π1)+π2(1−π2)π1−π2]2

### Data collection

Data were collected using personal-demographic, nutrition and physical activity questionnaires ( Supplementary File 1), the McGill Pain Index and height was measured by a metal ruler. The questionnaires were distributed by face to face interview. The personal-demographic questionnaire inquired about participants’ personal information, menstruation history, obstetric history and socio-economic status. The intervals of menstruation in a period of less than 21 days between 21 to 35 days or more than 35 days, according to the response of each person were marked. After obtaining the frequency, the mean of these were calculated in the two groups

The socio-economic status questionnaire contains 12 questions that were calculated using factor analysis method. Factor scores = 04754/0 * Education + 0/12080 * Assets + 0/34570 * Mother’s education + 0/27104 * Father’s education + 0/3585 * Type of home + 0/02277 * House size + 0/00403 * Number of residents At home - 0.06260 * Owning a private home 0/23442 * Mother’s income + 14.176 / 0 * Father’s income 0.04896 * Occupation. Using the above relationship, the socioeconomic score of each person was calculated.

The nutrition questionnaire consisted of 16 items that were scored based on a four-point Likert scale, with scores ranging from 16 to 64: never, 1; sometimes, 2; often, 3; always, 4. Questions 13 to 16 are never, 4; sometimes, 3; often, 2; always, 1. The nutrition questionnaire scores increased to a percentage and scores less than 33.3% of the total score of nutrition indicated poor nutrition, scores between 33.3% and 66.6% indicated somewhat proper nutrition and scores higher than 66.6% indicated good nutrition. Percentages were calculated as follows: Nutrition % = ((q1 + q2 + q3 + q4 + q5 + q6 + q7 + q8 + q9 + q10 + q11 + q12 + q13 + q14 + q15 + q16) - 16) / (64 - 16))* 100. The nutritional style questionnaire was used previously by Mahmoodi *et al.* for designing and psychometric evaluation. The Pearson correlation coefficient was 0.97. The Cronbach’s alpha coefficient in the nutrition aspect was 0.76, which confirmed its reliability and validity ^10^.

The physical activity dimension of participants’ lifestyle was assessed using the long-form International Physical Activity Questionnaire [IPAQ; http://youthrex.com/wp-content/uploads/2017/06/IPAQ-TM.pdf] ^11^, developed in 1998 by the WHO and CDD in Geneva as an international physical activity assessment tool for the age group 15 to 69. This version of the questionnaire consists of 27 items and reports physical activity levels in MET-minute/week and classifies people into three groups: A low activity group (less than 600 MET), a moderate activity group (between 600 and 3000 MET) and a high activity group (over 3000 MET) groups. The IPAQ is a global standard questionnaire whose validity and reliability have been approved in previous studies through content validity and Cronbach’s alpha ^12– 15^.

The McGill Pain Index is the most common visual analogue scale used in studies with an approved reliability and validity ^16^.

For data collection, the researcher (DAB) visited the study settings and obtained the permission of the directors of the centers. She conducted preliminary interviews with the participants (briefed them on the study objectives and the confidentiality of the data before they submitted their informed written consents). Eligible candidates were then selected for participation in the study.

### Data analysis

Data were analyzed in SPSS-18 using descriptive and analytical statistics such as mean and standard deviation, the independent t-test, the Chi-square test, Fisher’s Exact Test, Mann-Whitney’s U-test and the multiple logistic regression analysis.

### Ethical statement 

The study was conducted after obtaining the approval of the Ethics Committee of Shahid Beheshti University of Medical Sciences (ID: SBMU2.REC.1394.102). The authors obtained the consent of Mazandaran University of Medical Sciences for doing this research. Written informed consent was obtained from all the participants.

## Results

The results showed significant differences between the two groups in terms of age (P=0.001) and degree of education (P=0.011), but not in terms of BMI (p=0.296), age at menarche (p=0.374), duration of menstrual cycles (p=0.540) and intervals between menstrual cycles (p=0.054), which means that the two groups matched in terms of these four variables ( Table 1).

**Table 1.** Demographic, obstetric and gynecological characteristics of young women with and without primary dysmenorrhea.

Variables	Dysmenorrhea (n=125) Mean±SD	Without dysmenorrhea (n=125) Mean±SD	p-value
Age (years)	21.14±2.09	21.22±2.13	**0.001**
BMI	22.37±3.50	21.92±3.34	0.296
Menarch age (years)	13.39±1.39	13.24±1.30	0.374
Menstrual cycle	21.75±2.42	22.54±3.88	0.054
Duration of menstruation (days)	6.37±1.32	6.27±1.13	0.540
Socioeconomic status	4.24±0.904	4.13±0.877	0.346

In the group with dysmenorrhea, the good nutritional status was 21.6% and in the non-affected group it was 36%. According to the scores obtained in the questionnaires, the two groups were significantly different in terms of nutrition score (p=0.008) and physical activity (p=0.011); ( Table 2). The logistic regression analysis, however, showed no significant differences between the groups in terms of nutrition. The results showed a 1% reduction in the incidence of dysmenorrhea per each unit of increase in physical activity score; that is, a higher level of physical activity reduces the incidence of dysmenorrhea. Age also reduces the incidence of dysmenorrhea by 18%; in other words, the higher the age, the lower the incidence of dysmenorrhea ( Table 3).

**Table 2.** Frequency distribution and comparison of nutrition style and physical activity in young women with and without primary dysmenorrhea. Nutrition score was calculated using a four-point Likert score, while physical activity was calculated by MET (minutes/week).

Lifestyle characteristic	Dysmenorrhea (n=125) Mean±SD	Without dysmenorrhea (n=125) Mean±SD	p-value
Nutrition score	57.91±10.92	61.68±11.33	0.008
Physical activity (MET)	5518.75±3182/03	4666/42±1930/12	0.011

**Table 3.** Logistic regression model of effective factors on primary dysmenorrhea.

Lifestyle characteristic	Exp(B)=OR	Confidence interval (%95)	p-value	B
Age	1.208	1.040-1.404	**0.014**	0.189
Education	1.318	0.837-2.076	0.233	0.276
Nutrition score	0.977	0.951-1.004	0.089	-0.024
Physical activity	1.008	1.000-1.016	**0.040**	0.008

Raw data behind the results of this study The coding schema for the data can be found in Supplementary File 2. Click here for additional data file. Copyright: © 2018 Abadi Bavil D et al. 2018 Data associated with the article are available under the terms of the Creative Commons Zero "No rights reserved" data waiver (CC0 1.0 Public domain dedication).

## Discussion

The results showed that nutrition and physical activity were related to dysmenorrhea in the two groups. According to the results of Table 2, there was a significant difference between the two groups in terms of nutritional style (p = 0.008), physical activity (p = 0.11), but when some variables were adjusted by logistic regression analysis, nutrition didn’t show any difference between the two groups.

An optimal nutrition was found to reduce the severity of dysmenorrhea. In 1992, Ekstrom *et al.* ^17^ showed that, during menstruation, hypertonic saline infusion increases vasopressin and oxytocin, and along with the increase in these two hormones, the severity of dysmenorrhea also increases. Increased prostaglandin was proposed as the main reason for the pain and excessive bleeding experienced ^1^. Food items rich in magnesium can reduce the severity of dysmenorrhea by reducing the synthesis of prostaglandins and decreasing muscle and small vessel spasms ^18^. Following a high-fiber diet can increase sex hormone-binding globulins and thus reduce the synthesis of prostaglandins, which are the main cause of dysmenorrhea ^19^. Studies show that the arachidonic acid in animal fat is involved in the synthesis of prostaglandins, and therefore, foods such as meat and dairy are the main source of arachidonic acid ^5^. Regarding the link between the daily use of the four food groups and dysmenorrhea, it can be argued that the high consumption of fish, eggs, vegetables and fruits is associated with a low incidence of painful menstruation ^20^. Eliminating salty foods will decrease the incidence of dysmenorrhea as well ^21^. Having breakfast every morning ^22^ and eating nuts, pure honey ^24, 25^ are also effective in reducing the incidence of dysmenorrhea. The compound oleocanthal in extra virgin olive oil suppresses prostaglandin synthesis; in other words, it inhibits the enzymatic pathway for pain ^26^.

Exercise acts as a non-specific analgesia by improving pelvic blood circulation and stimulating the release of beta-endorphins ^9^. Exercise leads to the prevention and regression of dysmenorrhea by reducing stress and improving mood. Age at menarche is significantly higher in athletes ^27^. Exercise reduces body fat, and since obesity is associated with a high prevalence of dysmenorrhea, the loss of fat significantly increases age at menarche ^27^. Exercising three days before the beginning of the menstruation improves pelvic blood flow, disrupts the accumulation of prostaglandins in this part of the body and thus delays the onset of pain. Exercise during menstrual pain also leads to the faster transfer of excess substances and prostaglandin from the uterus, which is the main factor responsible for menstrual pain, and thus reduces the duration of pain during menstruation ^28^. Exercise can reduce the activity of the sympathetic nervous system and increase the activity of the parasympathetic nerves during rest and reduce stress and thereby menstrual symptoms ^29^. Regular aerobic exercise can reduce pain by increasing the secretion of endorphins, which are the most powerful natural opiates in the body ^30^.

Salehi *et al.* found a significant difference in the intensity and duration of pain after eight weeks of Pilates exercise between the intervention and control groups. On the first three days of menstruation, 30 minutes of brisk walking per day reduces primary dysmenorrhea pain. Dysmenorrhea was less prevalent in those who had regular exercise three sessions per week compared to those who did not exercise ^31^. Exercise is most effective in the prevention of dysmenorrhea when it begins before the first menstruation and remains a fixed part of the adult’s lifestyle ^27^. The present study showed that dysmenorrhea was less prevalent in those who were more physically active, and regular exercise can reduce stress in women and thus improve blood circulation and increase the amount of endorphins and neurotransmitters ^32^. Educational and counseling measures are needed to emphasize the importance of exercise.

The two groups were significantly different in terms of age. The prevalence of primary dysmenorrhea decreased with age. This condition is prevalent between ages 20 and 24 and then progressively declines in prevalence after this age ^33^. The two groups were not different in terms of BMI. Haidari *et al.* also showed no significant relationships between dysmenorrhea and the variables of BMI, height, weight and the waist-to-hip ratio ^34^. A positive relationship has been observed between a high BMI and dysmenorrhea. The inconsistency between the results obtained by Harlow ^35^ and those of the present study may be due to the fact that BMI is affected by factors such as race, age and gender and is therefore not a proper indicator of obesity, especially in athletes who have a high body mass ^31^.

In this study, no significant relationships were observed between the two groups in terms of age at menarche, the duration of menstrual cycles and intervals between menstrual cycles. Nevertheless, Espiroff found a significant relationship between age at menarche and the intensity of primary dysmenorrhea ^2^. The incidence of primary dysmenorrhea increases with longer intervals between menstrual cycles ^34^, heavy menstrual bleeding ^33^ and a menstruation lasting more than seven days ^36^. Chung *et al.* ^37^, however, argued that the duration of menstrual cycle is not related to dysmenorrhea. In the present study, the two groups were matched for confounding factors and there were therefore no differences between them in terms of menstruation history.

## Conclusion

Dysmenorrhea is a cyclical and debilitating process. Due to its negative impact on quality of life, preventive and supportive measures are necessary in young women by raising awareness and promoting education about better lifestyles, which encompass proper nutrition and regular physical activity.

## Data availability

The data referenced by this article are under copyright with the following copyright statement: Copyright: © 2018 Abadi Bavil D et al.

Data associated with the article are available under the terms of the Creative Commons Zero "No rights reserved" data waiver (CC0 1.0 Public domain dedication). 

Dataset 1: Raw data behind the results of this study. The coding schema for the data can be found in Supplementary File 2. DOI, 10.5256/f1000research.12462.d189275 ^38^

## Supplementary material

Supplementary File 1: Socio-demographic, nutrition and physical exercise questionnaires.

Supplementary File 2: Coding schema for Dataset 1.
