# Supplementary material for: A comparison of physical activity and nutrition in young women with and without primary dysmenorrhea
Source: F1000Res. 2018 Jan 16;7:59. [Version 1] doi: 10.12688/f1000research.12462.1 (PMC6117855; doi:10.12688/f1000research.12462.1)
Supplement: Supplementary file 2 [file f1000research-7-13494-s0001.tgz › 4318aa2d-4d8e-4370-92c6-4d002ada3c1f.docx]

Demographic profile questionnaire

1.age ...... .. years

2. Height .......

3. Weight ......

4. Body mass index ... ..

Menstrual records

5. Menarche (first menstruation) .....

6. The number of menstruation days ....

7. Between menstruation less than 21 days □ 21 to 35 days □ More than 35 days □

8. Anonymous □ Lar □ Fars □ Leave □

9. Are you required to follow a particular diet? Yes □ No □

10 If you are taking any vitamins, supplements, herbal remedies, sleep deprivation, sedative, etc., please indicate the amount and duration of use and the reason for its use. ...............................

11. My education ... ..

12. Mother's education ...... ..

 13. Father's education ......

14. Mother's job:

15. Father's job:

16. Do your household have the following items?

1. Electric vacuum cleaner 2. Separate kitchen 3. Computer Number: ...........

   4 Washing machine 5. Bath 6. Freezer

7. Dishwasher  8 .. Private car (not for work and monetization) Number: ........................ 9. mobile

10 Color TV 11. Types of video (VHS or VCD or DVD, etc.) 12. Landline (not mobile)

17. Your personal or family bank account: 1- Do not have 2

18. If yes, do you have the right to spend on your bank account: 1- Yes 2. Somewhat 3. No

19. Does your family have a steady income? 1-Yes 2- No

20 - Type of housing: Apartment house

21-Ownership: Tenant's tenant living with relatives

22-surface area of ​​the house (square meter) .....

23. Number of people living at home ......

24. Do you own a personal property in your name? 1- Yes 2- No

25. How did you get ownership of it? 1 - inheritance share 2 - gift from father 3 - purchase with personal income

26-Income

Own .... In the Mother's month .... in the month of Father .... in the month

Nutrition Style Questionnaire

| Always | Often | Sometimes | Never | This questionnaire contains your comments on daily nutritional behavior. Please respond to each item in detail. Try not to write an item. Specify the intensity of each behavior with the x sign. |
| --- | --- | --- | --- | --- |
|  |  |  |  | 1. I eat 2-4 pieces of fruit a day |
|  |  |  |  | 2. I use 3-5 vegetables a day |
|  |  |  |  | 3. I use 6-11 daily breads and cooks everyday |
|  |  |  |  | 4. Daily, 2-3 units of meat, beans and chicken. |
|  |  |  |  | 5. In cooking, I use more liquid liquids and use solid or animal oil at very low levels. |
|  |  |  |  | 6-.Only, I use nuts and oilseeds such as soya, sesame or sunflower seeds |
|  |  |  |  | 7 -I use more homemade food and eat less prepared foods like cans or a smaller meal |
|  |  |  |  | 8-Daily, 2-3 units of milk, yogurt or cheese |
|  |  |  |  | 9- I use a daily amount of sugar and I use it instead of dates or raisins. |
|  |  |  |  | 10-I will eat three meals a day |
|  |  |  |  | 11- I am eating enough food in my diet |
|  |  |  |  | 12-During the day, I use snacks like fruit, bread, cheese, or vegetables |
|  |  |  |  | 13-I do not use some sort of chips and pffk |
|  |  |  |  | 14- I do not use carbonated beverages |
|  |  |  |  | 15- I do not use salty foods |
|  |  |  |  | 16-I use less fried foods and I use to steam my food |

Physical activity questionnaire

We plan to get information about the types of physical activities people do as part of their everyday lives. The questions will be about the times that you have been active in the past 7 days. Please respond to any questions even if you do not consider yourself to be a full-time activist. Please consider all activities at work, as part of your work at home and in the building, to go from place to place, to activities that you perform as entertainment in leisure time and athletic exercises. Consider all the intense and moderate activities that you have taken over the past 7 days. Severe activities are said to be activities that require high levels of physical activity and make you breathe more quickly than normal. Moderate activity refers to activities that require moderate physical strength and make you feel a bit quicker than normal.

Section 1: Physical activity associated with daily work

The first part is related to your physical activity at the workplace, including salaried work, agriculture, voluntary work, periodic occupations, and any other non-salaried employment, except for unpaid work that you At home or outdoors, you take care of your home and your family (for example, work at home, work in the courtyard (garden), public utilities). These are discussed in Section 3.

Do you recently have jobs with salary or unlicensed rights (such as volunteering at a crescent)?

All 

No.  (Refer to Section 2: Shipping)

The next questions are about physical activity that you have done in the last 7 days as part of your paid or unpaid jobs. This does not include travel to and from the workplace.

During the last 7 days, how many days did it have intense physical activity, such as lifting heavy objects, digging, digging, heavy construction or climbing stairs as part of your car?

Consider only those physical activities that lasted for at least 10 minutes continuously.

.............. days a week

I have not had any severe physical activity. (Refer to question 4)

How often do you spend on such days to carry out these intense physical activities as part of your work?

................ hours per day

................. minutes a day

Again, consider the physical activity that lasts for at least 10 minutes continuously. During the last 7 days, how many days did it perform modest physical activities like carrying light loads as part of your work? Please do not count walking.

.............. days a week

I have not had a moderate physical activity activity (see Question 6)

How often do you spend on such days for these moderate physical activities as part of your work?

............. hours per day

.............. minutes a day

6. During the last 7 days, you have been walking for some 10 minutes as part of your work as part of your work for several days. Please do not count towards going to work or returning from work.

.............. days a week

I have not had a job related walk  (refer to section 2)

7. How often do you spend a lot of time on such days for walking as part of your work?

............. hours per day

.............. minutes a day

Section 2: Physical activity for transportation (commuting)

These questions relate to how you move from place to place (including places like work, stores, cinema, etc.).

8. During the last 7 days, how many days have you traveled with motor vehicles such as subway, bus, motorcycle or car?

.............. days a week

I have not been traveling with motor vehicles  (refer to question 10)

9. How often do you spend a lot of time on such days for travel by subway, bus, motorcycle, car or other motor vehicles?

............. hours per day

.............. minutes a day

Now consider things that bicycling or walking for transporting from place to place (traveling to or from a mission, etc.).

10. During the last 7 days, how many days have you cycled for a minimum of 10 minutes to go from place to place?

.............. days a week

I'm not going to be cycling elsewhere  (refer to question 12)

11 - How often do you spend a lot of time cycling to go from place to place?

............. hours per day

.............. minutes a day

12. During the last 7 days, how many days did you go to another place for a minimum of 10 minutes to go from place to place?

.............. days a week

I have not walked to a different location  (refer to section 3)

13. How often do you spend a lot of time on such days to go from one place to another?

............. hours per day

.............. minutes a day

Section 3: Homework, Home Improvement and Family Care

This section deals with activities that you have done inside or out during the last 7 days, such as homework, garden or courtyard work, public health and family care.

14- Take on physical activity for at least 10 minutes continuously. During the last 7 days, how many days did you have intense physical activity, such as carrying heavy loads, crushing wood, snow removal, or doing things like digging and plowing your garden in your garden or courtyard?

.............. days a week

I have not had any intense physical activity in the garden or courtyard of the house  (refer to question 16)

15. How often do you spend on such days for intense physical activities in the courtyard or garden of your home?

............. hours per day

.............. minutes a day

16- Consider the physical activities that you have been taking for at least 10 minutes continuously. During the last 7 days, how many days of moderate physical activity, such as carrying light loads, sweeping, cleaning windows, working with a fork (for collecting leaves, smoothing the surface of the soil, etc.) in the courtyard or garden?

.............. days a week

I have not had moderate physical activity in the courtyard or garden  (refer to question 18)

17. How often do you spend a lot of time on such days for these moderate physical activities in your yard or garden?

............. hours per day

............. minutes a day

18. Consider the physical activities that you have been taking for at least 10 minutes continuously. During the last 7 days, how many days of moderate physical activities, such as carrying light loads, cleaning windows, closing the floor and sweeping inside your home?

.............. days a week

Moderate physical activity inside the home  (refer to section 4)

19. How often do you spend a lot of time on these days for doing these moderate physical activities inside your home?

............. hours per day

............. minutes a day

Section 4: Recreation, Exercise and Physical Activity in Leisure

This section deals with activities that you have done in the last 7 days only for recreation, exercise, physical activity or leisure. Please do not consider the activities mentioned above.

20. Without regard to the walkways you have mentioned, during the last 7 days, how many days did you go for leisure for at least 10 minutes?

.............. days a week

I have not walked in my spare time  (refer to question 22)

21. How often have you walked on such days in your leisure time?

............. hours per day

.............. minutes a day

22. Take physical activity for at least 10 minutes continuously. During the last 7 days, how many days did you have intense physical activity such as aerobic exercise, running, fast biking, fast swimming, playing football in your spare time?

.............. I did not do any physical activity a day in my spare time  (refer to question 24)

23. How often do you spend a lot of time on such days for intense physical activity in your spare time?

............. hours per day

.............. minutes a day

24- Consider the physical activities that you have been taking for at least 10 minutes continuously. During the last 7 days, how many days did it have moderate physical activities such as average speed biking, medium speed swimming, two-person tennis (group), volleyball at your leisure?

.............. days a week

I have not had moderate physical activity in my spare time. (Refer to Section 5)

25. How often do you spend a lot of time on such days for doing moderate physical activity in your leisure time?

............. hours per day

.............. minutes a day

Section 5: Time spent sitting

These questions relate to times when you are sitting in a state of your own or in leisure at home or at work. This time can include the times you sit down or meet your friends and relatives, you study, sit or watch TV shows. Do not consider the times when you are seated in motor vehicles.

26. During the last 7 days, how often did you spend sitting in a typical working day?

............. hours per day

............... minutes a day

27. During the last 7 days, how often did you spend sitting on a weekend (weekend)?

............. hours per day

............... minutes a day
