# Supplementary material for: A comparison of physical activity and nutrition in young women with and without primary dysmenorrhea
Source: F1000Res. 2018 Jan 16;7:59. [Version 1] doi: 10.12688/f1000research.12462.1 (PMC6117855; doi:10.12688/f1000research.12462.1)
Supplement: Supplementary file 3 [file f1000research-7-13494-s0002.tgz › 19ce4c83-6666-451f-b4f2-558c640c444b.docx]

| Identification code | pain | age | height | weight | BMI | menarch age | bleeding |
| --- | --- | --- | --- | --- | --- | --- | --- |
| Participant identification code | intensity pain  0 to 10 | year | centimeter | Kilograms | Body mass index | year | the number of day bleeding |

**Supplementary File 2: Coding schema for Dataset 1.**

| Distance between menstruation | mother education | father education | mother Job | Father job |
| --- | --- | --- | --- | --- |
| 1=<21day  2=21-35day  3=>35day | 1=illiterate  2=Unfinished education  3=Diploma  4=Diploma  5=MA  6=P.H.D | 1=illiterate  2=Unfinished education  3=Diploma  4=Diploma  5=MA  6=P.H.D | 1=housewife  2=Free job  3=Employee  4=Retired  5=Doctor | 1= Unemployed  2=Free job  3=Employee  4=Retired  5=Doctor |

| Vacuum cleaner | kitchen | Computer | n. Computer | Clothes washer | Bathroom | freezer |
| --- | --- | --- | --- | --- | --- | --- |
| 0=no  1=yes | 0=no  1=yes | 0=no  1=yes | Number of computers at home | 0=no  1=yes | 0=no  1=yes | 0=no  1=yes |

| dish washer | car | n. car | Mobile | tv | dvd | telephone | Bank account |
| --- | --- | --- | --- | --- | --- | --- | --- |
| 0=no  1=yes | 0=no  1=yes | Number of cars at home | 0=no  1=yes | 0=no  1=yes | 0=no  1=yes | 0=no  1=yes | 1=yes  2=no |

| Pinch bank balance | Income of families | Home | Ownership of the house | Home meter | numbers of People living at home | Private house | How to own a home |
| --- | --- | --- | --- | --- | --- | --- | --- |
| 1=yes  2=some what  3=no | 1=yes  2=no | 1=House  2=the apartment | 1=the owner  2=Tenant  3=Living with relatives  4=Organizational Home | 1=,<100  2=101-200  3=201-300  4=>301 | Number of persons | 1=yes  2=no | 1=Inheritance  2=Gift  3=Personal |

| Individual monthly income | Mother's Monthly Income | Father's monthly income | job | physical activity in job (Ph.activity2) | D.ph.activity2 | t3 | moderate physical activity (ph.activity4) |
| --- | --- | --- | --- | --- | --- | --- | --- |
| 1=<500 thousand tomans  2=500 thousand tomans -1000000 Million  3=1-2milion  4=>2milion | 1=<500 thousand tomans  2=500 thousand tomans -1000000 Million  3=1-2milion  4=>2milion | 1=<500 thousand tomans  2=500 thousand tomans -1000000 Million  3=1-2milion  4=>2milion | 1=Employed  2=Non-employment | 1=yes  2=no | Number of days of intense physical activity related to work | Time spent on activity | 1=yes  2=no |

| D.ph.activity4 | t5 | Hiking related jobs (j.walking6) | D.J.walking6 | t7 | Use of motor vehicles | D.v.naghliye8 | t9 |
| --- | --- | --- | --- | --- | --- | --- | --- |
| Number of days of average physical activity related to work | Time spent on activity | 1=yes  2=no | Number of work-related walking days | Time spent on activity | 1=yes  2=no | Number of days of travel by motorized vehicles | Travel time with motor vehicles |

| have going to bicycle in week | D.bicycle 10 | t11 | have go to walking in week (walkig12) | D.walking12 | t13 | have severe physical activity in week (sever.ph) | D.sever.ph14 | t15 | have moderate physical activity in week in garden (moderate.ph.ga) | D.moderate.ph.g16 |
| --- | --- | --- | --- | --- | --- | --- | --- | --- | --- | --- |
| 1=yes  2=no | Number of cycling days to go somewhere | Time spent on activity | 1=yes  2=no | Number of days walking from place to place | Time spent on activity | 1=yes  2=no | Number of days of intense physical activity in the yard or garden | Time spent on activity | 1=yes  2=no | The number of days a week |

| t17 | have moderate physical activity in week in home (moderate.ph.h) | D.moderate.ph.h18 | t19 | Hiking in leisure | D.walking20 | t21 |
| --- | --- | --- | --- | --- | --- | --- |
| Time spent on activity | 1=yes  2=no | The number of days a week | Time spent on activity | 1=yes  2=no | Number of holidays in leisure | Time spent on activity |

| go arobic.running  swiming,football.severe | D.severe.ph22 | t23 | go arobic.running.swiming,football.moderate | D.moderate.ph24 | t25 | t26 | t27 |
| --- | --- | --- | --- | --- | --- | --- | --- |
| 1=yes  2=no | The number of days go arobic.running  swiming,football.severe | Time spent on activity | go arobic.running.swiming,football.moderate | The number of days a week | Time spent on activity | Seated time | Seated time in friday |

| Nutriation q1 | Nutriation q2 | Nutriation q3 | Nutriation q4 | Nutriation q6 | Nutriation q7 | Nutriation q5 | Nutriation q6 |
| --- | --- | --- | --- | --- | --- | --- | --- |
| 1=Never  2=sometimes  3=Always  4=mostly | 1=Never  2=sometimes  3=Always  4=mostly | 1=Never  2=sometimes  3=Always  4=mostly | 1=Never  2=sometimes  3=Always  4=mostly | 1=Never  2=sometimes  3=Always  4=mostly | 1=Never  2=sometimes  3=Always  4=mostly | 1=Never  2=sometimes  3=Always  4=mostly | 1=Never  2=sometimes  3=Always  4=mostly |

| Nutriation q7 | Nutriation q8 | Nutriation q9 | Nutriation q10 | Nutriation q11 | Nutriation q12 | Nutriation q13 |
| --- | --- | --- | --- | --- | --- | --- |
| 1=Never  2=sometimes  3=Always  4=mostly | 1=Never  2=sometimes  3=Always  4=mostly | 1=Never  2=sometimes  3=Always  4=mostly | 1=Never  2=sometimes  3=Always  4=mostly | 1=Never  2=sometimes  3=Always  4=mostly | 1=Never  2=sometimes  3=Always  4=mostly | 1=Never  2=sometimes  3=Always  4=mostly |

| Nutriation q14 | Nutriation q15 | Nutriation q16 |
| --- | --- | --- |
| 1=Never  2=sometimes  3=Always  4=mostly | 1=Never  2=sometimes  3=Always  4=mostly | 1=Never  2=sometimes  3=Always  4=mostly |

| pain | Bmi | Age at menarch | kode age | kode bleeding | Property | kode Property | Code of Nutrition |
| --- | --- | --- | --- | --- | --- | --- | --- |
| 1=No dysmenorrhea  2=dysmenorrhea | 1=<18.5  2=18.5-24.9  3=25-29.9  4=>30 | 1=<11  2=12-15  3=>16 | 1=18-20  2=21-23  3=34-26  3=>26 | 1=3-5  2=5-7  3=7-9  4=>9 | scores | 1=0-20  2=21-40  3=41-60  4=61-80  5=81-100 | 1=<%33/3  2=%33/3-%66/6  3=>%66/6 |

| workwalk | workwalkmet | workmoderate | workmoderatemet | worksever | worksevermet | totalwork |
| --- | --- | --- | --- | --- | --- | --- |
| Number of work-related walking days x t7 | metxdayxtime  3.3 xdayxtime | D.ph.activity4xt5  (Number of days of average physical activity related to work  X t5 ) | 4xdayxtime | Number of days of intense physical activity sever related to workxt3 | 8xdayxtime | workwalkmet+ workmoderatemet+ worksevermet |

| domesticsever | Domesticsever met | domesticmoderateyard | domesticmoderatermet | domesticmoderateinside | domesticmoderateinsidemet |
| --- | --- | --- | --- | --- | --- |
| Number of days of intense physical activity in the yard or garden xt15 | 5.5xdayxtime | The number of days a weekxt17 | 4xdayxtime | D.moderate.ph.h18  (The number of days a week)xt19 | 3xdomesticmoderateinside |

| totaldomestic | leisurewalk | leisurewalkmet | leisuresevermet | leisuremoderate | leisuremoderatemet |
| --- | --- | --- | --- | --- | --- |
| = domesticmoderateinsidemet | Number of holidays in leisure x t21 | 3.3x leisurewalk | The number of days go arobic.running  swiming,football.severe xt23 | D.moderate.ph24  (The number of days a week)xt25 | 4x leisuremoderate |

| totalleisure | Sit1 | Sit2 | sitmet | trans walk | transwalkmet |
| --- | --- | --- | --- | --- | --- |
| leisurewalkmet  + leisuresevermet  +  leisuremoderatemet | (t26 * 6 + t27 * 1) | (sit1 / 7) | (1.3 * sit1) | D.walking12  Number of days walking from place to placext13 | 3.3x trans walk |

| transcycle | transcyclemet | Trans car | Trans car met | totaltrans | totalwalk1 | totalmoderat1 |
| --- | --- | --- | --- | --- | --- | --- |
| Number of cycling days to go somewhere xt11 | Metxdayxtime  6xdayxtime | Number of days of travel by motorized vehicles  x Travel time with motor vehicles | 1.3xdayxtime | transwalkmet+ transcyclemet+ Trans car met | transwalkmet+ leisurewalkmet | = workmoderatemet+ domesticmoderatermet+ domesticmoderateinsidemet  + leisuremoderatemet |

| totalsever | ph1 | ph2 | ph2kode  Physical activity code | RECODE faliyat | faliyat |
| --- | --- | --- | --- | --- | --- |
| =leisuresevermet | totalwalk1+ totalmoderat1+ totalsever | ph1+ totaltrans | 1=<%33/3  2=%33/3-%66/6  3=>%66/6 | RECODE faliyat (Lowest thru 600=1) (600 thru 3000=2) (3000 thru Highest=3) | =(ph2 + sitmet) |

| mww | mwm | mws | tmw | mtm | mtd | mtw | tmt |
| --- | --- | --- | --- | --- | --- | --- | --- |
| workwalkmet/60 | workmoderatemet/60 | worksevermet  /60 | totalwork/60 | Transmashinmet/60 | transcyclemet/60 | transwalkmet/60 | totaltrans/60 |

| mds | mdmy | mdmh | tmd |
| --- | --- | --- | --- |
| Domesticsever met/60 | domesticmoderatermet  /60 | domesticmoderateinsidemet  /60 | totaldomestic/60 |

| mwl | msl | mml | tml | ms | faliyatfiziki | metsit | ph6 | kodeph6 |
| --- | --- | --- | --- | --- | --- | --- | --- | --- |
| leisurewalkmet | leisuresevermet | leisuremoderatemet | totalleisure/60 | Sit1/60 | faliyat=(ph2 + sitmet) | Sit2x7 | =(ph2 + sitmet) | 1=<%33/3  2=%33/3-%66/6  3=>%66/6 |
