# Supplementary material for: A comparison of physical activity and nutrition in young women with and without primary dysmenorrhea
Source: F1000Res. 2018 Jan 16;7:59. [Version 1] doi: 10.12688/f1000research.12462.1 (PMC6117855; doi:10.12688/f1000research.12462.1)
Supplement: Peer review history

# Peer review: Referee response for version 1

**DOI:** 10.5256/f1000research.13494.r37383

**Published:** 2018-08-29

**Authors:** Zeinab Hamzehgardeshi

**Affiliations:** 1 Sexual and Reproductive Health Research Center, Mazandaran University of Medical Sciences (MazUMS), Sari, Iran

The topic is very attractive. Dysmenorrhea is a common gynecological pain among young women.The healthy life style can reduce the severity of pain in the young women. This cross sectional study is well designed. The instruments were valid. The results can be helpful for designing an intervention study.

 The manuscript is suitable for indexing after the response to reviewers. Please revise based on the following comments:

**Title:**

 Indicate the study's design.

**Introduction:**

1. Please present previous studies in the field and clarify the research gap.
2. Please present the aim of the study (state the specific objectives) at the end of the discussion.

**Methods:**

1. Please clarify the validity and reliability of the Persian version of the questionnaires.
2. Please describe the setting and location of the study.
3. Please give the eligibility criteria, the sources, and the methods of selection of the participants.
4. Please clearly define all variables.
5. Describe any efforts to prevent potential bias.

**Discussion:**

1. Please discuss limitations of the study.
2. Please clarify the generalisability of the study results.

I have read this submission. I believe that I have an appropriate level of expertise to confirm that it is of an acceptable scientific standard.

# Peer review: Referee response for version 1

**DOI:** 10.5256/f1000research.13494.r30514

**Published:** 2018-02-07

**Authors:** Mark Jones

**Affiliations:** 1 School of Public Health, The University of Queensland, Herston, QLD, Australia

This article requires additional information to be included. Also some of the information provided needs clarification.

 In the abstract it is reported that dysmenorrhea is seen in almost 50% of women but in the introduction it is reported that overall prevalence is 60-90% in adolescent girls. This seems contradictory?

 The sentences in the abstract results “Physical activity was calculated by MET scale (minutes/week). This index measured the amount of consumed energy at the time of activity relative to that consumed at resting time” would fit better in the abstract methods.

 In the abstract please say how many of the 250 girls were in each comparison group.

 It appears that the cases had greater physical activity than the controls (cases: 5518.75 vs controls: 4666.42). Please clarify and interpret accordingly.

 In the abstract conclusion it says: “A healthier and more favorable nutrition style and more regular physical activity reduces the **severity** of dysmenorrhea in girls.” But the comparison is between cases and controls rather than a study of the effect of physical activity and nutrition on the severity of dysmenorrhea? Also this conclusion implies a causal effect but this study is a cross-sectional study of association?

 It mentions “proper nutrition” in the abstract conclusion. Does this mean some of the study participants were not getting proper nutrition?

 What were the actual questions used to determine exposure status (i.e. dysmenorrhea)?

 More information is required on the sample of participants included in the study. How many students were approached? How many participated? What were the reasons for any exclusions? How were the controls matched? The inclusion criteria for cases is not clear e.g. what does “painless (0 to 3) mean? What were the inclusion criteria for controls?

 What were the actual parameters assumed for the sample size calculation? E.g. what effect size was assumed?

 I could not follow the methods on how socioeconomic status and nutrition scores were obtained? Please clarify.

 In the data analysis section please clarify which test was used for which data.

 Were all the potential confounders included in the analysis? E.g. what about smoking, alcohol, and other drug use?

 In Table 2 I couldn’t understand what “3182/03” and “1930/12” meant? The education result was not included in table 2? Was there any missing data?

 Why were the variables reported in Table 3 included in the logistic regression? Did you use a multivariable model? Why is the result for nutrition score no longer “statistically significant”?

 Please interpret the main findings of the study in terms of clinical significance.

 Please include a section on limitations of the study including missing confounding variables, potential for selection bias, potential for exposure/outcome misclassification.

I have read this submission. I believe that I have an appropriate level of expertise to confirm that it is of an acceptable scientific standard, however I have significant reservations, as outlined above.

# Peer review: Referee response for version 1

**DOI:** 10.5256/f1000research.13494.r29899

**Published:** 2018-01-23

**Authors:** Narjes Bahri

**Affiliations:** 1 Department of Midwifery, Gonabad University of Medical Sciences, Gonabad, Iran

I read the paper carefully and find it good and suitable for publication. Only one point in my opinion needs  to be clarified before publication as following:

 The two group of study have named "case and control" although the type of study has mentioned:" comparative descriptive study". There is a conflict that must be clarified.

Recommendations for authors:

1. The type of study would be changed to: cross sectional study
2. The name of groups would be change to: with dysmenorrhea / and without dysmenorrhea

I hope my recommendation can improve the quality of the paper.

I have read this submission. I believe that I have an appropriate level of expertise to confirm that it is of an acceptable scientific standard.
